# Supplementary material for: Chromothripsis during telomere crisis is independent of NHEJ, and consistent with a replicative origin
Source: Genome Res. 2019 May;29(5):737–49. doi: 10.1101/gr.240705.118 (PMC6499312; doi:10.1101/gr.240705.118)
Supplement: Supplemental Material [file supp_gr.240705.118_Supplemental_file_1.zip › contigs/annotated_contigs/DB41/contig.3.DB41_length_1427_mean_cov_9.8465311843.docx]

**DB41_length_1427_mean_cov_9.8465311843**

```
TGCTCTTCTCCAAGGGTCTCTACTAGGTGCTCACAAAAAAGACTGGGGCAGGGCAAGAGAACTGAGAGACTCTGTATGTGGCACAAAGT
 >chr10:116748167-116748577 - E=2e-234
CACAGAATTCTCTGATACATGAGCCCTTCTCTTATGTGAAAGTCATAAGTTCAATGGGGAGAGGCCCAAGAAAAGATGCACTGCCTTTG

GTAGAAGGGTGAAAGCAAGAGCTGTCTGCCACTCGAGGAAGGGCAGAAAATGAGCTCAGCCCAGGATCTTGCACTGATACAAAGCAGAG

ATCTGCCACTACTGGAGAAGGGGCTGAATTACTGAGAAATCACTGGATTCCATCCACTCCTGGAGTCTAGGAGCTCAGGGCCCAAGACC

AAGGCTGGAGGATGAAAACTAAAAAAACACCCTCTTACTCCATGACAAGCTATA|C|AACAAGTTCCCACCCTTATTACTCATTTCCCA
                                                        >chr8:92396217-92396496 + E=1e-
AATAAACAATGCAGGTATATTAGTTTCCTGTTGCTGCTGTAATAAATTGCCATGAATTTAGTGGCTTAAAACAGCACGGATTTGTTATT
155 p=4e-02
TTACAGTTCTAGATGACAGAAGTCACTGAAATGAGTCTGGAGATAAAATCAAGTTTTGGCAGGGCTGCATTCCTTCTGGAGGGTCTAGA

GGAGAACTCATATCCTTGCCCTTTCCAACTTTCAGAGGCCATCTGTATTCCTTGGGTCATGGCCCTGTC|ATGTGATC|AGCAACAGTT
                                                                              >chr10:11
GATCACTTGGGGAGGTCAAAAAGAGCATGGGTCAGATTCCTTCTGTGGCACAGATATGAAGACTTGCTGAAATCTGAGCATGGAGGGAC
6747435-116748164 - E=0e+00
ACACTAAGGAAAAACCCTTCCACAATTCAGGCCTCACATGAATCACAAAGCGCAGCAGCCCACTCTTGCAGTGAATGGAAGTCTGGTGC

ATTCTAAGTATAGCATCAACAAATCCAAACCTAGTTCAGCCACTGACTAGACTGCCCCAACCTCCCTCCACACTAATGACTGGACACAA

AAAATGTGCCTATTTCTGGATAGAAGTATGACTTATCTAAGTTTATTTTACAGGCAATATTTGGCATACAATCAAGAAATACAAAACAC

ACAGACAAGAATGAAAAAAAGCTGTTAACAGACTCACAAAGCTATTAACAGACCTAGACTTGAATGTCAGAACTGTCAGACAAGGAATT

GAAAGTAACTATGACTACTATGTTAAAGGATTTCATGGAAAAGGTAGACAAAATGCAGCAAAAGATAAAAAATGTCAGCAGAGCTGGGT

GCAGTGCCTCATGCCAGTAATCTCAGCAATTGGGGAGGCTGAGGCAGGTGGATCACCTGAGGTCAGGAATTCAAGACCAGCCTGGCCAA

CATGGTGAAACCCTGTTTCCACTAAAAAAATACAAAAATTAGCCAGCCACGGTGGTGGGCACCTGTAATTCCAACTACTCGGGAGGATG

AGGCAGG
```
